# TLR4 phosphorylation at tyrosine 672 activates the ERK/c‐FOS signaling module for LPS‐induced cytokine responses in macrophages

**DOI:** 10.1002/eji.202250056

**Published:** 2023-05-01

**Authors:** James E.B. Curson, Liping Liu, Lin Luo, Timothy W. Muusse, Richard M. Lucas, Kimberley S. Gunther, Parimala R. Vajjhala, Rishika Abrol, Alun Jones, Ronan Kapetanovic, Katryn J. Stacey, Jennifer L. Stow, Matthew J. Sweet

**Affiliations:** ^1^ Institute for Molecular Bioscience (IMB) IMB Centre for Inflammation and Disease Research and Australian Infectious Diseases Research Centre The University of Queensland Brisbane Queensland Australia; ^2^ School of Chemistry and Molecular Biosciences (SCMB) and Australian Infectious Diseases Research Centre The University of Queensland Brisbane Queensland Australia; ^3^ Friedrich Miescher Institute for Biomedical Research Basel Switzerland

**Keywords:** inflammation, macrophages, pattern recognition receptors, post‐translational modification, toll‐like receptor 4

## Abstract

TLRs engage numerous adaptor proteins and signaling molecules, enabling a complex series of post‐translational modifications (PTMs) to mount inflammatory responses. TLRs themselves are post‐translationally modified following ligand‐induced activation, with this being required to relay the full spectrum of proinflammatory signaling responses. Here, we reveal indispensable roles for TLR4 Y672 and Y749 phosphorylation in mounting optimal LPS‐inducible inflammatory responses in primary mouse macrophages. LPS promotes phosphorylation at both tyrosine residues, with Y749 phosphorylation being required for maintenance of total TLR4 protein levels and Y672 phosphorylation exerting its pro‐inflammatory effects more selectively by initiating ERK1/2 and c‐FOS phosphorylation. Our data also support a role for the TLR4‐interacting membrane proteins SCIMP and the SYK kinase axis in mediating TLR4 Y672 phosphorylation to permit downstream inflammatory responses in murine macrophages. The corresponding residue in human TLR4 (Y674) is also required for optimal LPS signaling responses. Our study, thus, reveals how a single PTM on one of the most widely studied innate immune receptors orchestrates downstream inflammatory responses.

## Graphical abstract

The PRR TLR4 is post‐translationally modified via phosphorylation at tyrosine residues 672 and 749 in mouse macrophages to enable optimal LPS‐induced inflammatory responses. Y672 phosphorylation, via the adaptor proteins SCIMP and the SYK tyrosine kinase, initiates an ERK1/2 and c‐FOS signaling cascade that promotes selective inflammatory responses. In contrast, Y749 phosphorylation has a broader role, being required to maintain TLR4 protein levels.

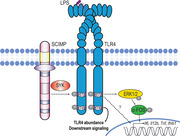

## Introduction

The innate immune system is tasked with sensing disruption of homeostasis, which occurs during injury and/or infection. Innate immune cells, such as macrophages, employ danger‐sensing PRRs [1] that recognize both exogenous pathogen‐associated molecular patterns and endogenous damage‐associated molecular patterns [2]. The TLR family of pattern recognition receptors is widely studied as a key orchestrator of innate immune inflammatory and antimicrobial gene expression programs. TLRs signal from both the cell surface and endosomal compartments, enabling them to recognize ligands derived from both extracellular and vesicular pathogens [3]. TLR4, a receptor for Gram‐negative bacterial LPS, is the most widely studied TLR family member [4, 5]. This receptor is unique within the TLR family, as it utilizes two sets of adaptor proteins to signal from both the cell surface and the endolysosomal compartment. TLR4‐mediated recognition of LPS is itself a complex process requiring multiple proteins. In the extracellular environment, LPS forms a complex with the secreted LPS‐binding protein. This complex is then recognized by CD14, which facilitates LPS loading onto a co‐receptor complex consisting of TLR4 and MD‐2 [6, 7]. This initiates the homodimerization of the TLR4 toll‐IL‐1 receptor (TIR) domain, promoting the recruitment of specific adaptor proteins to relay intracellular signaling.

The best characterized of the TLR4 adaptors are TIR domain‐containing proteins. TLR4 signaling involves two distinct temporal phases, with the initial response involving myeloid differentiation primary response protein 88 (MyD88)‐dependent signaling from the plasma membrane [8]. Subsequent to this, TLR4 internalization facilitates MyD88‐independent signaling from the endolysosomal compartment [9]. Upon ligand recognition, the first adaptor recruited to TLR4 is MAL, which then recruits MyD88 [10]. This clustering of TIR domains generates a stable platform upon which further MyD88 molecules can oligomerize into a complex known as the Myddosome [11]. The Myddosome consists of four to six MyD88, four IL‐1 receptor‐associated kinase (IRAK)4 and four IRAK2 molecules, acting as a signaling platform from which the IRAKs drive both MAPK and NF‐κB activation [12, 13]. These pathways culminate in the activation of numerous transcription factors, including NF‐κB, activating protein 1 (AP‐1), and IFN regulatory factor family members, thus, resulting in the expression of hundreds of immune‐related genes [14, 15]. Following ligand‐induced TLR4 homodimerzation at the cell surface, the receptor complex is internalized and MyD88‐independent signaling via TIR‐domain–containing adaptor‐inducing IFN‐β [16] and TIR‐domain–containing adaptor‐inducing IFN‐β(TRIF)‐related adaptor molecule (TRAM) is initiated [17]. These TIR‐containing adaptors continue to promote MAPK and NF‐κB signaling but also trigger tank binding kinase 1‐mediated phosphorylation of IFN regulatory factor3 (IRF3), enabling the expression of type‐one IFN genes and specific inflammatory mediators [9]. Tank binding kinase 1 also contributes to MyD88‐dependent signaling as part of the Myddosome, promoting LPS‐inducible glycolysis [18].

TLR4‐initiated signaling is relayed by a range of post‐translational modifications (PTMs), including serine or threonine phosphorylation, ubiquitylation, and lysine acetylation, of specific signaling molecules [19]. However, TLR4 [20, 21], along with its bridging adaptors MAL [22] and TIR‐domain–containing adaptor‐inducing IFN‐β (TRIF)‐related adaptor molecule (TRAM) [23], also undergoes tyrosine phosphorylation. A number of tyrosine kinases, including Bruton's tyrosine kinase [24], spleen tyrosine kinase (SYK) [25], and the Src family of tyrosine kinases [26], particularly Lyn [21, 27], have been implicated in the ligand‐dependent tyrosine phosphorylation of TLR4. Lyn binds a non‐TIR‐domain–containing adaptor protein called Slp65/76 and Csk‐interacting membrane protein (SCIMP) in murine immune cells [28], and we previously showed that Lyn and its kinase activity on SCIMP are required for an interaction between SCIMP and TLR4 in mouse macrophages [29, 30]. Consequently, SCIMP is required for optimal LPS‐inducible TLR4 phosphorylation, downstream signaling, and production of pro‐inflammatory cytokines in mouse macrophages [29]. More recently, we showed that the SYK tyrosine kinase binds to SCIMP, enabling the recruitment of SYK to TLR4 following LPS stimulation [31]. We also found that SYK is downstream of Lyn in this pathway [31], thus positioning SYK temporally and physically as the most proximal tyrosine kinase to TLR4 phosphorylation. Indeed, SYK is necessary for LPS‐induced TLR4 phosphorylation following LPS stimulation [31].

The specific mechanisms by which SCIMP enables TLR4 tyrosine phosphorylation, particularly the exact TLR4 tyrosine residue(s) involved, are currently unknown. Previous studies have delivered some insights into how specific TLR4 tyrosine residues regulate signaling responses, but they were often performed in artificial (nonimmune cell) systems, did not assess endogenous inflammatory outputs, and employed tyrosine to alanine (Y‐to‐A) mutants [21, 32, 33]. The latter is particularly important because Y‐to‐A mutations have the potential to affect protein structure and confound interpretation about the specific role of tyrosine phosphorylation. Here, we investigated the target(s) of SCIMP‐dependent TLR4 phosphorylation in primary mouse macrophages and the consequences of this on macrophage inflammatory responses. Our findings reveal a key role for TLR4 Y672 phosphorylation in driving proinflammatory cytokine responses.

## Results

### Identification of tyrosine residues in the TLR4‐TIR domain that are inducibly phosphorylated

**Figure 1:**
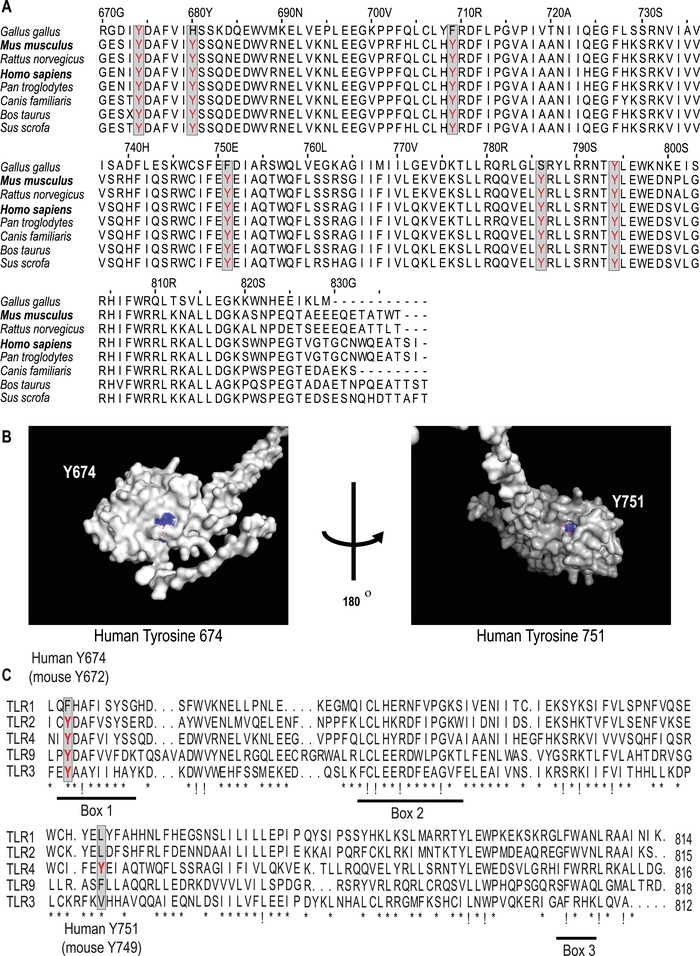
TLR4 tyrosine residues as candidates for phosphorylation. (**A**) The TIR domains of chicken, mouse, rat, human, chimpanzee, dog, cattle, and pig TLR4 were aligned via a clustal Omega multiple sequence alignment to identify conserved tyrosine residues within TLR4. (**B**) A prediction of the entire TLR4 structure was sourced from AlphaFold [34] and the TIR domain was visualized using Pymol. The presumed surface‐exposed tyrosine residues Y674 and Y751 are highlighted in blue. (**C**) The TIR domains of human TLR1, TLR2, TLR3, TLR4, and TLR9 were aligned via a clustal Omega multiple sequence alignment to identify conserved tyrosine residues within the TLR‐TIR domains. In the alignments, “!” indicates complete AA conservation and “*” indicates conservation of amino acids with strongly similar properties.

To identify candidate TLR4 tyrosine residues for regulated phosphorylation, we first used multiple sequence alignment to ascertain those residues that are conserved within the TIR domains of human, chicken, mouse, rat, human, chimpanzee, dog, cattle, and pig TLR4 (Fig. 1A). This analysis revealed six residues (human Y674, Y680, Y709, Y751, Y786, Y793, and their equivalents) that were conserved across almost all species, except for chicken (in which only Y674 and Y793 were conserved). Next, we used AlphaFold [34] to model full‐length human TLR4, identifying two of these TIR domain tyrosine residues (human Y674 and Y751) that are clearly predicted to be surface exposed (Fig. 1B). Given that SCIMP relays signaling downstream of multiple TLRs in murine cells [35], we next determined if these tyrosine residues are conserved across the murine TLR family. An alignment of TLR1, TLR2, TLR3, TLR4, and TLR9 revealed that Y674 (murine Y672) was conserved across all assessed TLRs, except TLR1 (Fig. 1C). In contrast, Y751 in human TLR4 (murine Y749) was only present in TLR4, although there are tyrosine residues in TLR1 and TLR2 that do not align perfectly with TLR4 Y751 but may be in spatially similar locations (Fig. 1C). We, therefore, predicted that Y674 might be involved in SCIMP‐dependent responses, whereas Y751 is likely to elicit its effects independently of SCIMP and to generate biological responses that may not necessarily be conserved with other TLRs.

**Figure 2:**
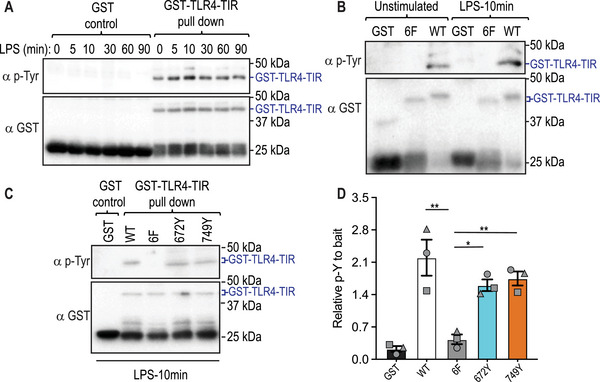
TLR4‐Y672 and Y749 are phosphorylated by extracts from LPS‐treated cells. (**A‐D**) A recombinant mouse TLR4‐TIR domain‐GST fusion protein was expressed in *E. coli*. GST‐TLR4‐TIR domains, alongside a GST only control, were purified prior to being incubated with lysates from RAW 264.7 cells that were stimulated with LPS (100 ng/mL) for the indicated time points. GST‐TLR4‐TIR domains were purified from the lysate mix and assessed for tyrosine phosphorylation via western blot. (**A**) Time‐dependent phosphorylation of the WT TLR4‐TIR domain. (**B**) WT GST‐TLR4‐TIR domain (WT) was compared alongside a mutant GST‐TLR4‐TIR protein in which six tyrosine residues (Y672F, Y678F, Y707F, Y749F, Y784F, Y792F) were mutated to phenylalanine (6F). (**C**) Tyrosine 672 (Y672) and 749 (Y749) were reintroduced into the 6F mutant and assessed, alongside WT and 6F TIR domains, for tyrosine phosphorylation upon incubation with lysates of LPS‐activated RAW 264.7 cells. (**D**) Quantification of western blots from panel (**C**) for total tyrosine phosphorylation, relative to total levels of relevant GST protein. Data are combined from three independent experiments (mean ± SEM, n = 3), with each symbol representing a different experiment. Statistical analyses were performed using a repeated measures one‐way ANOVA, followed by Bonferroni's multiple comparison test (*p < 0.05, **p < 0.01).

We previously showed that LPS‐inducible tyrosine phosphorylation on TLR4 requires SCIMP [29], but we did not identify the tyrosine(s) involved. In human cell lines, both LPS [36] and EGF [37] triggered TLR4 phosphorylation at Y674 (mouse Y672 equivalent), highlighting this residue as a candidate for SCIMP‐mediated phosphorylation. We attempted to independently verify the LPS‐inducible phosphorylation of mouse TLR4 at Y672 and Y749, using IP coupled to MS to directly assess TLR4 tyrosine phosphorylation in RAW 264.7 murine macrophage‐like cells expressing ectopically TLR4. These attempts were unsuccessful, likely due to the very low levels of TLR4 expression in cells, even when overexpressed from a strong EF‐1α promoter. We, therefore, sought an alternative approach to assess the phosphorylation of mouse TLR4. To this end, we expressed the TIR domain of mouse TLR4 (GST‐TLR4‐TIR) in *Escherichia coli*, then treated the purified GST‐TLR4‐TIR with cell lysates from LPS‐activated RAW 264.7 cells and assessed total tyrosine phosphorylation. This analysis confirmed the LPS‐inducible phosphorylation of the TLR4‐TIR domain at 5–10 min post‐stimulation (Fig. 2A), although there was also considerable phosphorylation of TLR4 after incubation with non‐stimulated cell extracts under these conditions. Next, all the tyrosine residues in the TLR4‐TIR protein, identified in Fig. 1A, were mutated to phenylalanine (6F). Treatment of this GST mutant with lysate from LPS‐activated RAW 264.7 cells showed no signal in the anti‐phosphotyrosine blot, as expected (Fig. 2B). When Y672 or Y749 were reintroduced into the 6F mutant, thus creating TLR4‐TIR domains that contain only a single tyrosine of the six tyrosines highlighted in Fig. 1A, there was a return of TLR4‐TIR domain tyrosine phosphorylation (Fig. 2C & D). This indicates that both residues are phosphorylated, although the response was not completely restored to that of the WT GST‐TLR4‐TIR. We note that pulldown of TLR4‐TIR domains exposed to LPS‐activated lysates results in the appearance of a protein of variable abundance approximately 5 kDa above the expected TLR4‐TIR band (Fig. 2A‐C, upper band in anti‐phosphotyrosine blots that runs just below 50 kDa). This may represent a modified or alternatively translated tyrosine‐phosphorylated form of the TLR4‐TIR domain or a tyrosine‐phosphorylated protein that interacts with the TLR4‐TIR domain.

### Phosphorylation on Y749, but not Y672, is required for optimal TLR4 protein levels

**Figure 3:**
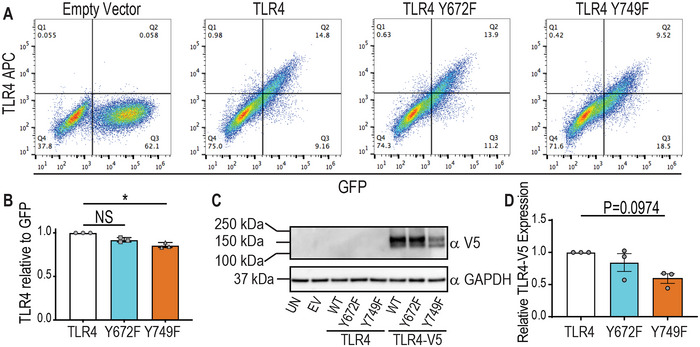
(**A**) **TLR4 Y749F, but not a Y672F, mutation results in decreased levels of TLR4 protein**. (**A, B & D**) *Tlr4^−/−^* BMM were reconstituted with WT mouse TLR4, the indicated TLR4 mutants or empty vector (EV). (**C**) *Tlr4^−/−^* BMM were reconstituted with V5‐tagged WT mouse TLR4, the indicated V5‐tagged TLR4 mutants or empty vector (EV). (**A**) Cells were assessed for transduction efficiency (plasmid coding for GFP) and surface TLR4 (APC anti‐TLR4 antibody) via flow cytometry. (**B**) The total number of APC (TLR4)‐positive cells was quantified as a ratio to the total number of GFP‐positive cells and plotted relative to the levels in BMM transduced with WT TLR4. (**C**) Whole cell lysates were collected and assessed for total TLR4 expression via western blot for α‐V5 (UN = untransduced control, EV = empty vector transduced control, WT = WT TLR4). (**D**) Western blots were quantified for total TLR4‐V5 expression (relative to GAPDH) and plotted relative to levels in BMM transduced with WT TLR4. (**A & C**) Data are representative of three independent experiments. (**B** & **D**) Data are combined from three independent experiments (mean ± SEM, n = 3) and statistical analyses were performed using a Kruskal–Wallis test, followed by Dunn's multiple comparison test (NS, non‐significant, *p < 0.05).

To functionally investigate Y672 and Y749 in mouse TLR4 responses, we generated retroviral expression constructs with tyrosine to phenylalanine substitutions for each residue (Y672F, Y749F), then expressed them in *Tlr4^−/−^* murine BM‐derived macrophages (BMM). When we assessed TLR4 surface expression in these cells, the TLR4‐Y749F‐expressing cells displayed reduced cell‐surface TLR4 when compared to those cells transduced with WT TLR4, despite the total percentage of transduced cells being similar (Fig. 3A & B). Consistent with this, we also observed by immunoblotting that in *Tlr4^−/−^* BMM transduced with V5‐tagged TLR4 constructs, TLR4‐Y749F‐V5‐expressing BMM had reduced total TLR4 protein compared to those expressing WT TLR4‐V5 (Fig. 3C & D). In contrast, the TLR4‐Y672F‐V5 mutant was expressed at similar levels to WT TLR4‐V5 (Fig. 3C & D). The decrease in TLR4 Y749F protein expression was not a consequence of differences in mRNA expression upon retroviral transduction, as both TLR4‐Y672F‐ and TLR4‐Y749F‐expressing cells had *Tlr4* mRNA levels comparable to cells transduced with WT TLR4 (**Supporting Information** Fig. S1A). We note that some *Tlr4* mRNA was still detectable in the *Tlr4^−/−^* BMM (empty vector control) as these mice were generated through the targeted deletion of the region encoding amino acids 86–835 [5] and our qPCR primers recognize nucleic acids outside this region. We conclude that phosphorylation at Y672 does not affect either cell‐surface or total TLR4 expression. In contrast, phosphorylation at Y749 likely controls TLR4 protein levels, potentially by limiting TLR4 degradation. We note that SCIMP silencing did not affect total TLR4 levels in BMM [29], so these findings are consistent with SCIMP acting via phosphorylation of Y672, rather than Y749.

### Y672 and Y749 phosphorylation does not affect LPS‐mediated downregulation of surface TLR4

**Figure 4:**
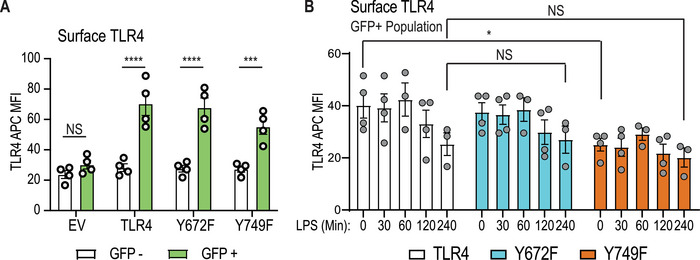
**TLR4 Y672 and Y749 are not essential for LPS‐inducible TLR4 internalization**. *Tlr4^−/−^* BMM were reconstituted with WT mouse TLR4, the indicated TLR4 mutants or empty vector (EV). (**A**) The median fluorescence intensities of the TLR4 signal in unstimulated cells were compared between GFP^–ve^ (untransduced) and GFP^+ve^ (transduced) populations. (**B**) Cells were treated for the indicated time with 10 ng/mL LPS and assessed for cell‐surface TLR4 via flow cytometry. Within the GFP^+ve^ population, the median fluorescence intensities of each TLR4‐expressing population are presented following the subtraction of the median fluorescence intensity of EV (*TLR4^−/−^*) samples. (**A & B**) Data (mean ± SEM, n = 3–4) are combined from three to four independent experiments. Statistical analyses were performed using a repeated measures two‐way ANOVA (**A**) or a mixed‐effect analysis (**B**), followed by Bonferroni's multiple comparisons test (NS: nonsignificant, *p < 0.05, ***p < 0.001, ****p < 0.0001).

We next examined whether either mutation affects LPS‐mediated downregulation of TLR4 surface expression as readout of TLR4 internalization. When comparing cell‐surface TLR4 expression of GFP^+ve^ and GFP^−ve^ populations in transduced cells, we observed that TLR4 expression at the cell surface was reconstituted in the GFP^+ve^ population, as expected (Fig. 4A). In these GFP^+ve^/TLR4^+ve^ cells, cell‐surface TLR4 was reduced at the basal state in BMM reconstituted with Y749F but not Y672F (Fig. 4B), consistent with previous observations (Fig. 3B). However, LPS treatment still caused some reduction in levels of cell‐surface TLR4 in cells expressing the Y749F mutant, with these levels being similar to those of WT TLR4 and the Y672F mutant at 240 min post‐LPS stimulation (Fig. 4B). These data suggest that phosphorylation at either Y749 or Y672 does not affect TLR4 internalization in mouse macrophages.

### TLR4‐Y672 and ‐Y749 are required for optimal inflammatory cytokine responses

**Figure 5:**
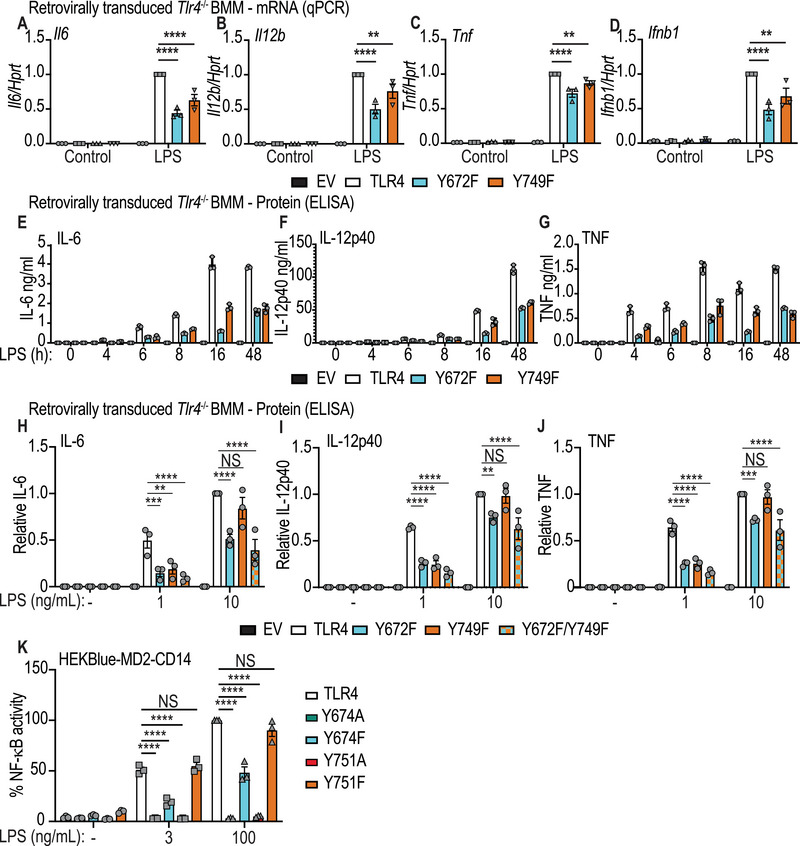
TLR4 Y672 and Y749 are essential for optimal LPS‐inducible cytokine production. (**A‐I**) *Tlr4^−/−^* BMM were reconstituted with WT mouse TLR4, the indicated TLR4 mutants or empty vector (EV). Cells were then treated with 1 ng/mL LPS (**A‐G**) or the indicated LPS concentrations (**H‐K**). (**A‐D**) Cells were stimulated with LPS for 4 h before total RNA was collected and assessed by RT‐qPCR for mRNA levels of *Il6*, *Il12b*, *Tnf*, and *Ifnb1*. Data (mean + SEM, n = 3) are combined from three independent experiments and are normalized to the WT TLR4 + LPS sample. (**E‐G**) Cells were stimulated with LPS for indicated times, after which supernatants were collected and assessed for levels of IL‐6, IL‐12p40, and TNF by ELISA. Data (mean + range, n = 3) are technical replicates from one experiment, with similar observations made in two independent experiments. (**H‐J**) Cells were stimulated with the indicated concentrations of LPS for 24 h and supernatants were collected and assessed for levels of IL‐6, IL‐12p40, and TNF via ELISA. Data (mean + SEM, n = 3) are combined from three independent experiments and are normalized to the WT TLR4 + LPS 10 ng/mL sample. (**K**) HEKBlue cells stably expressing CD14 and MD‐2 and with an integrated NF‐κB mScarlet reporter were transiently transfected with human TLR4, TLR4 Y674A, TLR4 Y674F, TLR4 Y751A, or TLR4 Y751F expression constructs, and then stimulated with LPS at the indicated concentrations for 16 h. Cells were then harvested and assessed for NF‐κB‐dependent reporter activation via flow cytometry. The mScarlet geometric mean fluorescence activity was collected from cells gated on low detectable levels of TLR4‐GFP protein and each condition was normalized to the WT TLR4 + LPS 100 ng/mL sample and represented as a percentage. Data (mean ± SEM, n = 3) are combined from three independent experiments. (**A‐K**) Statistical analyses were performed using a repeated measures two‐way ANOVA, followed by Bonferroni's multiple comparison test (**p < 0.01, ***p < 0.001, ****p < 0.0001).

We next assessed the capacity of TLR4‐Y672F and ‐Y749F mutants to promote LPS‐inducible inflammatory cytokines. Here, we found that both mutants produced significantly less LPS‐inducible *Il6*, *Il12b*, *Tnf*, and *Ifnb1* mRNAs after retroviral transduction in *Tlr4^−/‐^* BMM, by comparison to WT TLR4 (Fig. 5A‐D). LPS‐inducible mRNA levels of *Il12a* and *Il23a*, which encode IL‐12 cytokine family members, were also reduced (**Supporting Information** Fig. S1B & C). In contrast, inducible expression of *Ebi3* that encodes another IL12 cytokine family member, as well as the chemokine‐encoding genes *Ccl2* and *Cxcl2*, were all unaffected by either mutation (**Supporting Information** Fig. S1D‐F). These data suggest that Y672 and Y749 phosphorylation have selective effects on downstream inflammatory responses. Consistent with the gene expression data, LPS‐inducible IL‐6, IL‐12p40, and TNF protein secretion in response to a submaximal LPS concentration (1 ng/mL) were substantially reduced over a time course (Fig. 5E& G). When examining inducible cytokine production at submaximal (1 ng/mL) versus maximal stimulatory (10 ng/mL) LPS concentrations, the TLR4‐Y672F‐expressing cells showed a statistically significant reduction in the inducible production of IL‐6, IL‐12p40, and TNF in both conditions (Fig. 5H‐J). However, the defect in TLR4‐Y749F‐expressing cells was only apparent in cells stimulated with 1 ng/mL LPS. This is consistent with a model in which Y749 phosphorylation regulates TLR4 protein levels, rather than selective signaling responses. That is, the level of TLR4 is not the rate limiting factor in the response once a maximal stimulatory concentration of ligand is used. In BMM expressing a double mutant of both tyrosine residues (TLR4‐Y672F/Y749F), secreted levels of LPS‐inducible IL‐6, IL‐12p40, and TNF in response to submaximal LPS concentrations were consistently lower than either of the single mutants, although the effect was not statistically significant (Fig. 5H‐J).

To determine whether the cytokine defect in the TLR4‐Y749F cells responding to submaximal LPS concentrations (Fig. 5H‐J) was likely a consequence of TLR4 expression levels, we turned to a reporter‐based system that enables comparison of signaling responses in cells expressing similar matched levels of receptors. HEK293T cells stably expressing CD14 and MD‐2, and with an integrated NF‐κB‐dependent mScarlet reporter construct, were transiently transfected with either WT human TLR4 tagged with GFP, or the corresponding tyrosine mutants of mouse TLR4 (human Y674A, Y674F, Y751A, and Y751F). LPS‐inducible mScarlet expression was then assessed in cell populations expressing equivalent amounts of TLR4‐GFP protein. Here, we observed that substitution of either residue with alanine ablated LPS‐inducible NF‐κB‐dependent reporter activity, whereas this response was attenuated in cells expressing Y674F but not Y751F (Fig. 5K). These data confirm that phosphorylation of Y672/Y674 (mouse/human) is required for maximal proinflammatory signaling responses and are consistent with reduced TLR4 protein expression being responsible for the defects observed in mouse BMM expressing Y749F and responding to submaximal LPS concentrations (Fig. 5A‐J).

### TLR4‐Y672 phosphorylation triggers the ERK/c‐FOS signaling module

To identify mechanisms that may be linked to defective inflammatory responses in BMM expressing Y672F, we next investigated acute TLR4 signaling in cells responding to a submaximal LPS concentration (as seen in Fig. 5). In keeping with the effect of the Y749F mutation on basal TLR4 expression, LPS‐inducible phosphorylation of NF‐κB (p65 subunit), phosphorylation of the MAPKs (ERK1/2, JNK, p38), and TBK‐1 (**Supporting Information** Fig. S2A‐I) were all diminished to varying degrees in cells transduced with the Y749F construct, by comparison to WT TLR4. Neither Y672F‐ nor Y749F‐expressing cells had any discernible change in the total protein expression of any of the assessed signaling molecules (**Supporting Information** Fig. S2B). Despite the pronounced impact of the Y672F mutation on cytokine outputs (Fig. 5A‐J), this mutation generally had more modest effects on signaling responses, with only a minor reduction in JNK and p38 phosphorylation being observed over the time course (**Supporting Information** Fig. S2C & D). The exception to this was a trend toward enhanced AKT phosphorylation (**Supporting Information** Fig. S2F). Collectively, these data do not reveal a strong candidate signaling module by which Y672 phosphorylation exerts its proinflammatory effects.

**Figure 6:**
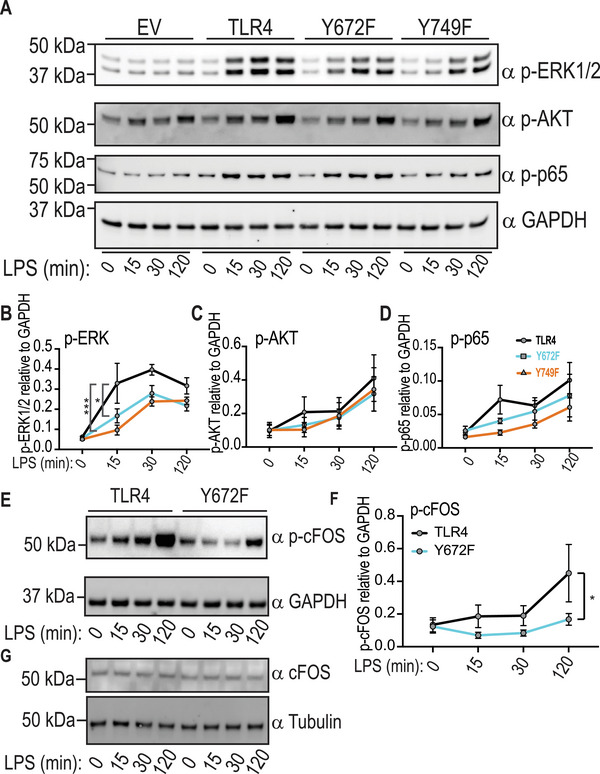
**TLR4 Y672F‐expressing macrophages display impaired ERK1/2 phosphorylation. (A‐G)** *Tlr4* ^−/−^ BMM were reconstituted with WT TLR4, TLR4 mutants or empty vector (EV). Cells were washed of CSF‐1 and left in media for 4 h before treatment with LPS (1 ng/mL) for the indicated time points. Whole cell lysates were collected and assessed by western blot for levels of phosphorylated or total proteins, as indicated. (**B‐D & F**) Western blots were quantified for protein expression (relative to levels of GAPDH). (**A & E**) Data are representative of three independent experiments. (**G**) Data are representative of two independent experiments. (**B‐D & F**) are combined from three independent experiments. Statistical significance in (**B‐D & F**) was determined using a repeated measures two‐way ANOVA, followed by a Bonferroni's multiple comparison test (*p < 0.05, ***p < 0.001).

Colony stimulating factor‐1 (CSF‐1) activates both ERK1/2 [38, 39] and AKT [40, 41], so examining TLR‐regulated ERK1/2 and AKT signaling in CSF‐1‐replete BMM can be complicated by the fact that these pathways are constitutively active in these cells (see **Supporting Information** Fig. S2A). To examine CSF‐1‐independent phosphorylation events downstream of TLR4 activation, retrovirally transduced BMM were plated in the absence of CSF‐1 for 4 h prior to stimulation with LPS. In this setting, TLR4‐Y672F transduced cells displayed a pronounced reduction in ERK phosphorylation (Fig. 6A & B). The phosphorylation of AKT and p65 was also slightly reduced, although in this case, the effects were not significant (Fig. 6A, C & D). Again, TLR4‐Y749F‐expressing cells displayed reduced phosphorylation of all signaling molecules that were assessed (Fig. 6A‐D), consistent with a broader role for Y749 in controlling levels of TLR4 protein.

We next investigated how Y672 phosphorylation‐dependent ERK1/2 activation might contribute to inflammatory responses. ERK1/2 promotes TLR‐inducible cytokine production by phosphorylating the AP‐1 transcription factor component c‐FOS [42, 43, 44]. We recently showed that c‐FOS also mediates SCIMP‐dependent TLR responses in macrophages [45]. Here, we found that c‐FOS phosphorylation (Fig. 6E & F) was markedly reduced in cells expressing Y672F, while total c‐FOS levels remained unchanged (Fig. 6G). These data support a model in which Y672 phosphorylation enables optimal ERK1/2 activation, leading to c‐FOS phosphorylation and inducible expression of inflammatory genes.

### SCIMP and SYK act via TLR4 Y672 for LPS‐inducible cytokine production

**Figure 7:**
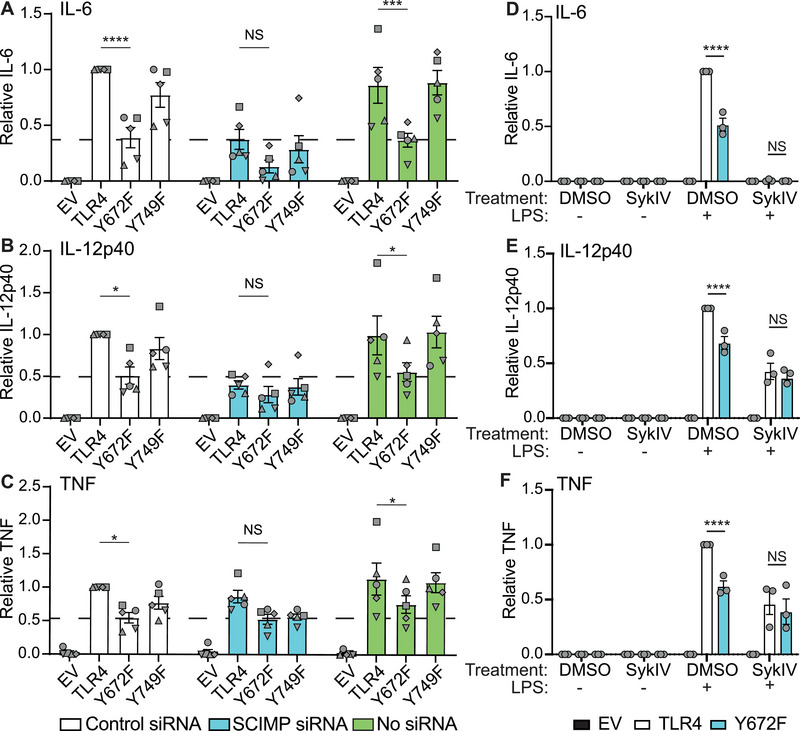
Role of SCIMP and SYK in TLR4 Y672‐dependent cytokine responses. (**A‐F**) *Tlr4* ^−/−^ BMM were reconstituted with WT mouse TLR4, TLR4 mutants or empty vector (EV). (**A‐C**) Cells were electroporated with *Scimp* siRNA, a control siRNA or no siRNA, after which cells were treated with LPS (10 ng/mL) for 24 h. Supernatants were collected and assessed for levels of IL‐6, IL‐12p40, and TNF by ELISA. Dashed lines indicate cytokine levels in Y672F‐expressing BMM transfected with control siRNA to facilitate comparison between conditions. Data (mean + SEM, n = 5) are combined from five independent experiments with different symbols showing data points from each experiment and are normalized to the control siRNA WT TLR4 sample. (**D‐F**) Transduced cells were preincubated with 10 μM of the SYK‐specific inhibitor SykIV for 30 min, then stimulated with LPS (10 ng/mL) for 24 h. Supernatants were collected and assessed for levels of IL‐6, IL‐12p40, and TNF by ELISA. Data (mean + SEM, n = 3) are combined from three independent experiments and are normalized to the WT TLR4 DMSO + LPS‐treated sample. (**A‐F**) Statistical analyses were performed via a repeated measures two‐way ANOVA, followed by Bonferroni's multiple comparison test (NS: non‐significant, *p < 0.05, **p < 0.01, ****p < 0.0001).

Since SCIMP scaffolds tyrosine kinases for LPS‐inducible TLR4 phosphorylation [29, 31], we next determined if SCIMP lies upstream of Y672. To do so, *Scimp* was silenced in *Tlr4^−/−^* BMM expressing WT TLR4, TLR4‐Y672F, or TLR4‐Y749F, after which LPS‐inducible cytokine production was assessed. As expected, SCIMP depletion (**Supporting Information** Fig. S2J) substantially reduced LPS‐inducible production of IL‐6 and IL‐12p40 downstream of WT TLR4 but had a less pronounced effect on TNF by comparison to the no siRNA and control siRNA transfections (Fig. 7A‐C). SCIMP depletion in cells transduced with WT TLR4 resulted in LPS‐inducible cytokine levels that were very similar to the two control populations (no siRNA, control siRNA) that had been transduced with TLR4 Y672F (Fig. 7A‐C). Hence, the Y672F mutation largely phenocopies *Scimp* silencing in BMM. Moreover, the reduction in inducible IL‐12p40 production caused by the Y672F mutation was less pronounced in *Scimp*‐silenced cells, by comparison to the control cell populations. In contrast, the Y749F mutant did not phenocopy *Scimp*‐silenced WT TLR4‐expressing BMM. Considering that SCIMP silencing attenuates LPS‐inducible TLR4 phosphorylation [29], these data support a model in which SCIMP promotes phosphorylation at Y672 to activate ERK1/2 and c‐FOS, driving inflammatory cytokine production.

We recently found that LPS promotes recruitment of the tyrosine kinase SYK to SCIMP and that targeting either SYK or SCIMP results in a similar profile of cytokine inhibition [31]. Having identified Y672 phosphorylation as a possible link between SCIMP and activation of the Erk/c‐FOS signaling module, we next examined the potential involvement of SYK in Y672‐dependent inflammatory responses. Cells transduced with TLR4 or TLR4‐Y672F were pretreated with the SYK‐specific inhibitor BAY‐61‐3606 (SykIV) [46] for 30 min prior to LPS stimulation. SYK inhibition abolished IL‐6 production (Fig. 7D) and attenuated the production of IL‐12p40 and TNF in cells expressing WT TLR4 (Fig. 7E & F). However, SYK inhibition did not further reduce the production of these mediators in cells expressing TLR4‐Y672. While the IL‐6 data (Fig. 7D) indicate that SYK likely has broad effects beyond the SCIMP‐TLR4 axis, the IL‐12p40, and TNF data (Fig. 7E & F) are consistent with SYK‐mediated phosphorylation of Y672. We, therefore, conclude that the SCIMP/SYK axis drives phosphorylation of TLR4, likely on Y672, with this permitting activation of ERK1/2 and c‐FOS to drive proinflammatory gene expression in macrophages.

## Discussion

Historically, most of the focus on TLR4 signaling has been on the PTMs of downstream effectors, rather than on TLR4 itself. Previous studies did investigate roles for specific TLR4 tyrosine residues [21, 32, 33], but these studies were not performed in immune cell populations and did not specifically address the role of tyrosine phosphorylation. In this study, we demonstrate that the TLR4‐TIR domain is both basally and LPS‐inducibly phosphorylated. We have shown for the first time that Y672 and Y749 residues on recombinant TLR4 are directly phosphorylated, that these PTMs are necessary for optimal TLR4‐mediated inflammatory responses in murine macrophages, and that distinct mechanisms are involved for each of these tyrosine residues. Primary macrophages expressing TLR4‐Y749F had reduced levels of total TLR4 protein, with a concomitant reduction in both TLR4 signaling and cytokine outputs in response to stimulation with submaximal LPS concentrations. In contrast, TLR4‐Y672F‐expressing BMM displayed a significant decrease in LPS‐inducible cytokine production in response to both maximal and sub‐maximal LPS concentrations, likely due to a defect in engagement of the ERK1/2 and c‐FOS signaling module that lies downstream of SCIMP [29] and the SCIMP‐associated tyrosine kinase SYK [31].

Previous studies showed that mutating Y674 to an alanine in human TLR4 ablated NF‐κB activation and promoter‐reporter activity in HEK293 cells [21, 32, 33]. The contribution of Y751 on human TLR4 to LPS responses has also been investigated in HEK293‐based systems, with dual mutations encompassing this residue (YE‐751/752 to AA‐751/752) resulting in the loss of NF‐κB, C/EBP, AP‐1, IL‐12p40, and IL‐10 promoter‐reporter activity [33]. Consistent with this, we also found that human Y674A and Y749A‐TLR4 mutants were unable to drive LPS‐inducible NF‐κB‐dependent reporter activity (Fig. 5K). However, such defects could arise from structural changes in the TLR4‐TIR domain or loss of hydrophobic interactions and may not have any relevance to the role of tyrosine phosphorylation. Indeed, human TLR4‐Y674F and Y751F mutants were permissive of LPS‐inducible NF‐κB signaling (Fig. 5K). This was also the case for the equivalent mouse TLR4 mutants (Y672F, Y749F) with respect to both signaling (Fig. 6A‐F) and cytokine production (Fig. 5A‐J). However, these mutants were significantly impaired for LPS‐inducible cytokine responses, particularly the Y672F mutant (Fig. 5H‐J). Collectively, these data confirm that Y672 and Y749 phosphorylation are required for optimal TLR4 responses in mouse macrophages and suggest that the more pronounced phenotypes of tyrosine to alanine mutations in previous studies of human cells [21, 32, 33] are likely due to effects on overall TLR4 protein structure or on interactions with other amino acids through pi‐stacking.

A previous study showed that a TLR4 Y674A mutant constitutively associated with MyD88 in HEK293 cells, whereas its LPS‐inducible interaction with this adaptor protein was attenuated by comparison to that of WT TLR4 [21]. These previous findings might suggest that phosphorylation of TLR4 Y672 enhances the binding of TLR4 to TIR‐domain containing adaptor proteins. However, such alterations would be predicted to result in more severe signaling defects than were observed here (**Supporting Information** Fig. S2). The selectivity of both SCIMP [45] and Y672 phosphorylation (Fig. 6A) in promoting ERK1/2 phosphorylation suggests a more compartmentalized role for this specific PTM in the TLR4 signaling response. Our data would support a model in which phosphorylation on Y672 functions to selectively enhance downstream signaling, rather than acting as an on and off signaling switch.

We recently showed that SCIMP scaffolds ERK1/2 and presents it to TLR4, enabling LPS‐inducible ERK1/2 and c‐FOS phosphorylation [45]. Our observation that TLR4‐Y672F‐expressing BMM had a similar effect on LPS‐inducible signaling (Fig. 6A‐F) suggests a role for SCIMP in promoting Y672 phosphorylation. This is further supported by our finding that SCIMP knockdown broadly phenocopies Y672F‐dependent cytokine release, particularly for IL‐12p40 (Fig. 7A & B). ERK1/2 has established roles in both pro‐ and anti‐inflammatory cytokine production downstream of TLRs, with the upstream kinase MAP3K tumor progression locus 2 linked to these effects [47, 48]. The bifurcation of these ERK1/2‐dependent responses is likely performed through confinement of ERK1/2 to specific cellular compartments, for example, via SCIMP‐mediating scaffolding at the cell surface for proinflammatory responses [45]. The data presented here suggest that the activation of ERK1/2 for proinflammatory responses involves TLR4‐Y672 phosphorylation. Perhaps TLR4‐Y672 phosphorylation stabilizes the TLR4‐SCIMP interaction, facilitating a transfer of ERK1/2 from SCIMP to TLR4, compartmentalizing ERK1/2, and enabling its activation at the cell surface. We recently identified an alternatively translated form of SCIMP, SCIMP translational variant 1 (SCIMP TV1), which lacks the first 13 amino acids at the *N*‐terminus [49]. In contrast to full‐length SCIMP, SCIMP TV1 has a distinct intracellular localization and selectively promotes CpG DNA responses in murine macrophages [49]. Given that TLR9 has a conserved tyrosine residue in a similar position to that of Y672 in TLR4 (Fig. 1C), it is possible that SCIMP TV1 may similarly compartmentalize CpG DNA‐induced ERK1/2 activation proximal to TLR9.

While our observations suggest that Y672 phosphorylation has a selective role in TLR4 signaling via ERK1/2 and c‐FOS, it is likely that this PTM on TLR4 also has consequences outside of c‐FOS and AP‐1 activation. For example, we found that Y672 phosphorylation is required for maximal LPS‐induced *Ifnb1* mRNA expression, whereas a previous study reported that ERK1/2‐TPL2 and c‐FOS suppress this response in macrophages [50]. Further, investigation of both Y672‐dependent and ‐independent inflammatory outputs, such as those observed in genes encoding members of the IL‐12 cytokine family (**Supporting Information** Fig. S1B‐D), may reveal such Y672‐dependent signaling modules. We also observed modest decreases in JNK and p38 phosphorylation 2 h post‐LPS stimulation in Y672F‐expressing cells (**Supporting Information** Fig. S2A, C‐D). This could reflect a direct effect of the Y672F mutation on acute TLR4 signaling or it could be a consequence of reduced autocrine signaling downstream of TLR4‐induced inflammatory cytokine mediators.

In TLR4 Y749F mutant‐expressing BMM, the reduction in total TLR4 protein levels (Fig. 3C & D) and LPS responsiveness (Fig. 5E‐H) indicate a unique role for Y749 phosphorylation. Given the decrease in TLR4 protein levels (Fig. 3C & D) and cell‐surface TLR4 (Fig. 4B) in unstimulated TLR4 Y749F‐expressing cells, it is likely that this residue is phosphorylated in the basal state. For example, it may be phosphorylated during TLR4 folding and anterograde transport, possibly influencing its stability or rate of turnover at the cell surface. Previous studies have also revealed that the tyrosine kinase SYK constitutively associates with TLR4 [25, 51, 52] and that more SYK protein is recruited to TLR4, following LPS stimulation in human monocytes [25, 51–54]. Both pharmacological inhibition [31] and gene silencing [55] of SYK impaired TLR4‐dependent inflammatory cytokine release, so it is likely that SYK binding to TLR4 is a mechanism integral to both basal and ligand‐inducible TLR4 phosphorylation, enabling downstream inflammatory cytokine production.

We previously observed both basal and LPS‐inducible TLR4 phosphorylation in BMM, with siRNA‐mediated *Scimp* depletion ablating LPS‐inducible TLR4 phosphorylation [29]. More recently, we characterized the SCIMP‐SYK interaction, finding that Y96 on SCIMP, which is necessary for its association with TLR4 [29], is also a docking site for one of the two SH2 domains of SYK [31]. Our findings thus imply that the proinflammatory actions of SYK and SCIMP are partially reliant on their ability to promote TLR4 phosphorylation, presumably through Y672 phosphorylation. Although the SCIMP‐bound tyrosine kinase Lyn is also implicated in the phosphorylation of TLR4 [21], and thus, represents another candidate, we recently showed that SYK lies downstream of Lyn in LPS signaling [31]. This is also consistent with findings from others, in which SYK was downstream of Lyn in activated innate immune cells [54, 56]. Moreover, our previous work directly implicated SYK in TLR4 phosphorylation [31]. Thus, SYK presents as the most likely candidate mediating TLR4 phosphorylation at Y672 and downstream inflammatory signaling responses. Nonetheless, SCIMP‐bound proteins have complex interdependences, and we have yet to fully decipher the molecular events both preceding and succeeding TLR4‐TIR domain phosphorylation.

**Figure 8:**
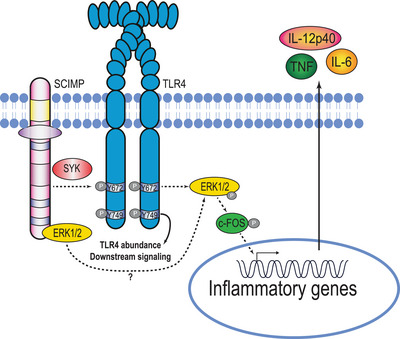
Updated model of TLR4 phosphorylation and its downstream effects. TLR4 Y749 phosphorylation is required for maintenance of TLR4 abundance and overall signaling competency. SCIMP scaffolds both SYK and ERK1/2 in the TLR4 signaling complex, likely enabling both the LPS‐inducible phosphorylation of TLR4 Y672, as well as subsequent activation of ERK1/2 and c‐FOS, to drive inflammatory cytokine production.

In conclusion, we demonstrate that phosphorylation of both Y672 and Y749 are necessary for optimal TLR4 signaling in murine macrophages. Our findings support a model in which Y672 phosphorylation, likely via SCIMP and SYK, enables activation of ERK1/2‐ and c‐FOS‐dependent proinflammatory cytokine outputs. In contrast, Y749 phosphorylation has a unique role in maintaining TLR4 protein abundance and downstream signaling (Fig. 8). A greater understanding of these two molecular events may ultimately deliver new approaches to dampen inflammation in TLR4‐driven diseases.

## Materials and methods

### Animal handling

All animal studies were reviewed and approved by the appropriate University of Queensland animal ethics committee. *Tlr4^−/‐^* mice, which were originally generated on a 129/Svj background [5], were backcrossed more than 10 times on to a C57BL/6 background at the Queensland Biosciences Precinct animal house at the University of Queensland, where these mice were housed for these studies.

### Cell culture and reagents

BMM were generated from femur and tibia BM extracted from 8‐ to 12‐week‐old male and female C57BL/6 mice. Post‐euthanasia, tibias and femurs were collected and sterilized using 70% ethanol prior to flushing of the BM with a 27‐gauge needle (Terumo). Once extracted, BM from all four bones was cultured in eight square 10 cm^2^ bacteriological plastic culture dishes (formerly Sterilin, Thermo Fisher Scientific) for 6–7 days in the presence of recombinant human CSF‐1, used at either 1 × 10^4^ U/mL (Chiron) or 150 ng/mL (The University of Queensland Protein Expression Facility), as previously described [57]. BMM were cultured in RPMI1640 (Gibco) supplemented with 2 mM Glutamax (Life Technologies), 10% heat‐inactivated FBS, 50 U/mL penicillin, and 50 μg/mL streptomycin (BMM complete media). BMM were harvested on day 6 and plated in BMM complete medium for experimentation on day 7, unless described otherwise. Platinum‐E retroviral packaging (PlatE) cells [58] were cultured in the presence of DMEM (Gibco) containing 2 mM l‐glutamine and supplemented with 10% FBS, 50 U/mL penicillin, and 50 μg/mL streptomycin (PlatE complete media). RAW 264.7 cells were cultured in RPMI1640 supplemented with 2 mM glutamax, 5% FBS, 50 U/mL penicillin, and 50 μg/mL streptomycin (RAW complete media). HEK293‐derived cells were maintained in DMEM supplemented with 2 mM glutamax, 10% FBS, 50 U/mL penicillin, and 50 μg/mL streptomycin (all reagents from Life Technologies). LPS from *Salmonella enterica* serotype Minnesota Re 595 (L2137, Sigma–Aldrich) was used at concentrations listed in individual figures, except for reporter assays on HEK293‐derived cells that used *E. coli* LPS (tlrl‐3pelps, Invivogen). The SYK inhibitor SykIV, also known as BAY61‐3606 (Merck), was dissolved in DMSO to a concentration of 2 mg/mL and stored at −20°C, before it was diluted in relevant media to be used at a concentration of 10 μM. Polybrene (Merck) was dissolved in ultrapure water to a concentration of 10 mg/mL.

### Mammalian expression vectors

A murine TLR4 expression construct was purchased from Addgene (plasmid # 13085; http://n2t.net/addgene:13085; RRID:Addgene_13085). The *Tlr4* cDNA was subcloned from pcDNA3.1 into pEF6_V5/HisTopo via PCR. Y672F and Y749F mutations were introduced into two different constructs via PCR mutagenesis using primers described in **Supporting Information** Table S1. Primers not incorporating a stop codon were used to generate V5‐tagged variants of all three constructs. All constructs were then subcloned via restriction enzyme digest and ligation into the retroviral expression construct pMIGRMCS_GFP [59], which has an internal ribosome entry site, thus, permitting both TLR4 and GFP expression from the same transcript. TLR4 Y672/749F double mutants were synthesized by Gene Universal using TLR4_pMIGRMCS_GFP as a template. Human *TLR4* was ligated to eGFP and inserted into pcDNA3 via restriction enzyme digest. Human *TLR4* mutants were made using Q5 (New England Biolabs) or Quikchange (Stratagene) site‐directed mutagenesis using primers described in **Supporting Information** Table S1. All constructs generated are summarized in **Supporting Information** Table S2 and were confirmed by automated DNA sequencing (AGRF).

### Gene overexpression by retroviral transduction in BMM

A total of 2 × 10^6^ PlatE [58] cells were plated in 10‐cm dishes and left to adhere overnight. PlatE cells were transiently transfected with empty vector pMIGRMCS_GFP or specific TLR4_pMIGRMCS_GFP constructs (**Supporting Information** Table S2) using lipofectamine 2000 (Invitrogen). At 24 h post‐transfection, cells were washed and incubated at 32°C for 48 h to facilitate virus production. At the same time, mouse BM was collected from *Tlr4^−/−^* mice, plated in BMM complete media, and incubated at 37°C for 48 h. A total of 10 mL of viral supernatant was collected from transfected PlatE cells, filtered for retrovirus using 0.45 μm Millex‐HV PVDF syringe filter (Merck) into labeled 15 mL Falcon tubes. A total of 20 mM HEPES (Gibco), 60 ng polybrene, and 10^4^ U CSF‐1 were added to each supernatant. BMM progenitors were collected from plates and added equally to retroviral supernatants, before being aliquoted into nontissue culture six‐well plates. Plates were centrifuged at 1000*g* at 35°C for 2 h to facilitate viral uptake. At 48 h post‐infection, media was replaced with BMM complete media. BMM were collected on day 6 and assessed for transduction efficiency by measuring GFP expression by flow cytometry, before being plated for further experiments.

### TLR4‐TIR GST protein expression and pull‐down assays

Codon‐optimized mouse WT TLR4‐TIR (AAs: 670–835) and indicated mutants were all subcloned into the pGEX6p‐1 vector. The GST‐tagged recombinant proteins were then expressed in *E. coli* BL21 (DE3) cells. Briefly, 2 μL of bacterial glycerol stock was inoculated into 500 mL of Lutia–Bertani media with ampicillin (100 μg/mL) and incubated overnight at 37°C with shaking (200 rpm). The overnight starter culture was then incubated in 20 L of media with antibiotics to amplify bacterial production. *Escherichia coli* cultures were grown for approximately 2.5 h to reach the mid‐exponential phase (OD_600_ = 0.8–1.0). At this time, isopropyl β‐d‐thiogalactoside was added at a final concentration of 0.4 mM for 4 h at 37°C to induce protein expression. Cell pellets were then harvested by centrifugation at 6000*g* for 10 min and were resuspended on ice in the lysis buffer (50 mM Tris‐HCl, 150 mM NaCl, 0.5 mM PMSF, pH 7.5). Cells were disrupted by a high‐pressure homogenizer for two cycles with a pressure of 30 KPSI. For the enrichment of GST‐TLR4‐TIR and mutant proteins, cell lysates were captured by glutathione sepharose beads and were directly used as bait for pull‐down experiments.

For the detection of TLR4‐TIR phosphorylation, TLR4‐TIR recombinant proteins that were immobilized on glutathione beads were coincubated with lysates from LPS‐induced macrophage‐like RAW 264.7 cells. GST‐coupled beads were used as a negative control. Specifically, 20 × 10^6^ cells were plated in P30 plates in 20 mL of RAW complete media. After overnight culture, cells were treated with LPS (100 ng/mL) for the time points described in figures. Cells were then lysed with chilled lysis buffer (25 mM Tris, pH 7.4, 150 mM NaCl, 1% NP‐40, 1% Triton 100, cOmplete Mini EDTA‐free protease inhibitor cocktail tablets [Sigma‐Adrich, Australia], 5% glycerol and PhosSTOP [Roche Applied Science, Switzerland]). The cell lysates were then incubated with bait‐bound sepharose beads on a roller at 4°C for 3 h. After incubation, the beads were washed three times with ice‐cold lysis buffer before being eluted in 2 × SDS‐PAGE sample buffer. Eluted proteins were heated at 95°C for 10 min for immunoblotting analysis.

### Whole cell extracts and immunoblotting

Whole lysates were collected by lysing cells in radioimmunoprecipitation assay buffer (50 mM Tris pH 7.4, 150 mM NaCl, 1 mM EDTA, 1% Triton X‐100, 1% sodium deoxychalate, 0.1% SDS) supplemented with cOmplete™, EDTA‐free protease inhibitor cocktail (Sigma) and PhosSTOP phosphatase inhibitor (Sigma). Immunoblotting was performed by electrophoresing equal amounts of protein (determined by bicinchoninic acid assays) through precast BOLT gels (Invitrogen), followed by turbo transfer at 25 V for 9 min onto nitrocellulose membranes (BioRad). Membranes were then blocked in 5% BSA in tris‐buffered saline containing 0.05% TWEEN^®^ 20, followed by probing with the indicated antibodies (**Supporting Information** Table S3). Proteins were visualized using Clarity ECL (BioRad) and Chemidoc. Membranes were either stripped using ReBlot Plus Strong Solution (Merck) at RT for 15 min or quenched with hydrogen peroxide 30% (Merck) at 37°C for 20 min, prior to reprobing of blots.

### Quantification of cell surface and total cellular TLR4 by flow cytometry

A total of 1 × 10^6^ transduced BMM were plated on six‐well plates and left to adhere overnight. The media was removed, and cells were collected using 1 mL of chilled lift buffer (PBS containing 0.1% sodium azide and 2 mM EDTA) and kept on ice for the duration of the experiment. Samples were washed two times with 1 mL chilled FACs buffer (3% BSA filtered in PBS), then blocked in 50 μL of FACs buffer containing FcX Trustrain (BioLegend) to reduce antibody nonspecificity. Antibody diluted in FACs buffer was added directly to the blocked sample (TLR4 allophycocyanin [APC], BioLegend SA15‐21, 2.5 μg/mL) and incubated on ice and in the dark for 1 h. The antibody was then removed, after which samples were washed twice with 1 mL chilled FACs buffer before being washed and then resuspended in PBS. Samples were analyzed for GFP expression and antibody staining (APC) using a Gallios flow cytometer (Beckman Coulter) or a Fortessa flow cytometer (BD Bioscience).

### ELISA

Levels of secreted mouse IL‐6, IL‐12p40, and TNF were assessed via sandwich ELISA using antibodies listed in **Supporting Information** Table S3. A 96‐well ELISA plate (Nunc) was coated with capture antibody (diluted in 0.1 M sodium bicarbonate, pH 8.35) overnight. Plates were washed twice with PBS containing 0.05% tween (PBST), before being blocked with 10% FBS in PBS for 2 h at 37°C, or overnight at 4°C. Plates were washed before samples and standards (diluted in the relevant complete media) were added and incubated for 2 h at 37°C or overnight for 4°C. Plates were then sequentially incubated and washed with secondary antibody (diluted in 10% FBS in PBS) for 1 h at 37°C, followed by extra‐avidin (1:1000 dilution in 10% FBS in PBS) for 20 min at 37°C. After further washing, TMB substrate (BD OptEIA) was added. Reactions were stopped using 2 M sulfuric acid and absorbance at 450 nm was read using a plate reader (Infinite M Plex, Tecan). Cytokine levels were calculated by extrapolation from a sigmoidal curve analysis of the standards.

### NF‐κB reporter assays for TLR4 signaling

HEK‐Blue^TM^ hMD2‐CD14 cells (Invivogen) stably expressing an NF‐κB‐mScarlet‐I reporter [60] were plated at 64,000 cells per well in a 96‐well plate. The transfection complexes were formed with 200 ng *hTLR4*‐eGFP expression plasmids and Lipofectamine 2000 (Life Technologies) according to the manufacturer's instructions. To maximize transfection efficiency, the complexes were added to the cells and centrifuged at 700*g* for 10 min before overnight incubation [61]. Transfection medium was replaced with fresh DMEM medium containing 5% FBS, after which cells were incubated for 6 h. Cells were then left untreated or were treated with 3 or 100 ng/mL LPS overnight. Samples were analyzed via a BD Cytoflex flow cytometer for GFP (excitation at 488 nm and emission at 525 nm) and mScarlet‐I (excitation at 561 and emission 585) expression. The mScarlet‐I geometric mean of cells gated on those with detectable low TLR4‐GFP level is displayed relative to the level seen in cells transfected with a construct encoding WT TLR4 and treated with 100 ng/mL LPS.

### siRNA‐mediated gene silencing

siRNA knockdown of *Scimp* was performed as previously described [35]. Day 6 BMM were harvested, cells were resuspended in complete media at a concentration of 4 × 10^6^ cells/350 μL, and 10 μL 1 M HEPES (tissue culture grade) per milliliter media was added. Cell suspensions (350 μL) were transferred to 0.4 cm electroporation cuvettes and mixed with siRNAs against *Scimp* or *Ctr2/Dnm1* (control gene) to a final concentration of 0.5 μM or tissue culture grade water (no siRNA control) in a final volume of 400 μL. Cells were electroporated at 240 V, 1000 μF, and ∞ Ω. After electroporation, cells were washed twice, counted, and then plated at the required cell numbers. Cells were treated with indicated stimuli at 24 h post‐transfection. Sequences of siRNAs used were: mScimp #1: sense sequence: 5′‐AGACAACCCUCAGCUUGGUACUCAU‐3′; antisense sequence: 5′‐AUGAGUACCAAGCUGAGGGUUGUCU‐3′; control (mCtr2 #1): Sense Sequence: 5’‐UCUCAGAUGAGGCCGUGCUUCUCUU Antisense Sequence: 5’‐AAGAGAAGCACGGCCUCAUCUGAGA or control (mDnm1 #1): MSS203618.

### MTT assays

To assess plating density, in concurrence to any experiment where an ELISA was performed, 4 × 10^4^ cells (from the same working stock used to plate cells for analysis of cytokine production by ELISA) were plated in 96‐well plates and left to adhere overnight. Cells were incubated with 1 mg/mL MTT reagent (Sigma) and diluted in the appropriate complete media. Cells were left at 37°C for 1–3 h. MTT media was removed and formazan crystals were dissolved in 100% isopropanol. Once the formazan precipitate was fully dissolved, the absorbance at 510 nm was read using a plate reader (Infinite M Plex, Tecan).

### RNA purification and cDNA synthesis

Cells were lysed in 350 μL TRIzol (Invitrogen) or RLT buffer (QIAGEN), after which total RNA was extracted using the relevant RNA extraction kit (Zymo for TRIzol, QIAGEN for RLT), as per the manufacturer's guidelines. Total RNA was quantified using a ND1000 nanodrop spectrophotometer (Thermo Fisher Scientific). Contaminating genomic DNA was removed using on‐column DNAse digestion (Qiagen) during RNA extraction. A total of 1000 ng of RNA was incubated at 65°C for 5 min in a cocktail containing oligo dT primers (Merck) and 10 mM dNTP, followed by 1 min incubation on ice. The RNA/oligo dT dimer was then reverse transcribed using a cocktail containing Superscript III, first strand reaction buffer and 0.1 M DTT (Invitrogen), with incubation at 50°C for 50 min then 70°C for 10 min. A no‐RT control was generated using RNA collected for all samples in a set that was then treated as above, but without the incorporation of Superscript III into the RT cocktail. cDNA samples were diluted in ultrapure DNAse/RNAse‐free water (Gibco) and stored at −20°C.

### Gene expression analysis via RT‐qPCR

RT‐qPCR was performed in 384‐well plates (Applied Biosystems), with each well containing 5 μL SYBR Green PCR Master Mix (Applied Biosystems), a total of 1 μL of forward and 1 μL of reverse primers at 2 μM (**Supporting Information** Table S4), a total of 1 μL of DNAse/RNAse‐free water (Gibco), and 2 μL of diluted cDNA. All samples were run in triplicate wells for each gene of interest, and levels of mRNA were quantified in a 7900HT fast RT‐PCR system (Applied Biosystems). Gene expression was normalized to the expression of the housekeeping gene hypoxanthine phosphoribosyltransferase *(Hprt*) and analyzed using the delta *Ct* method [62].

### Statistical analyses

Quantitative data acquired from each independent experiment were averaged across technical replicates, after which data from independent experiments were combined and represented as the mean ± SEM of n (n = number of independent experiments). Data with *n* < 3 were represented as the mean ± range of the data. Statistical analyses on data combined from ≥ 3 independent experiments were performed using GraphPad Prism**©** software, using statistical tests that are described in individual figure legends. These include repeated measures one‐way, two‐way ANOVA followed by a Bonferroni *post‐hoc* multiple comparisons test, mixed‐effect analysis followed by a Bonferroni *post‐hoc* multiple comparisons test, or the Kruskal–Wallis test followed by Dunn's multiple comparisons test.

## Conflict of Interest

The authors declare that they have no commercial or financial conflict of interest with the contents of this article.

## Author contributions

James E. B. Curson: Conceptualization, Methodology, Investigation, Visualization, Writing—Original Draft. Liping Liu: Investigation, Methodology, Writing—Review & Editing. Lin Luo: Conceptualization, Investigation, Supervision, Methodology, Writing—Review & Editing. Timothy W. Muusse: Investigation, Resources. Richard M. Lucas: Conceptualization, Writing—Review & Editing. Kimberley S. Gunther: Investigation. Parimala R. Vajjhala: Resources, Writing—Review & Editing. Rishika Abrol: Methodology, Investigation. Alun Jones: Methodology, Investigation. Ronan Kapetanovic: Supervision, Conceptualization. Katryn J. Stacey: Conceptualization, Supervision, Resources, Writing—Review & Editing. Jennifer L. Stow: Conceptualization, Supervision, Writing—Review & Editing, Funding acquisition. Matthew J. Sweet: Conceptualization, Supervision, Writing—Original Draft, Writing—Review & Editing, Funding acquisition, Project administration.

## Ethics approval

All animal studies were reviewed and approved by the appropriate University of Queensland animal ethics committee under approval numbers IMB/121/15/ARC/NHMRC/BREED, IMB/118/18/BREED, IMB/118/15/ARC/NHMRC, IMB/123/18 and 2021/AE000629, and 2021/AE000630.

### Peer review

The peer review history for this article is available at https://publons.com/publon/10.1002/eji.202250056

**Abbreviations**
AP‐1: Activating protein 1
APC: Allophycocyanin 
BMM: BM‐derived macrophages
IRAK: IL‐1 receptor‐associated kinase
MyD88: Myeloid differentiation primary responses protein 88
PTMs: Post‐translational modifications
SCIMP: Slp/Csk‐interacting membrane protein
SYK: Spleen tyrosine kinase
TIR: Toll/Interleukin 1 receptor
TV1: Translational variant 1

## Supporting information

Supporting information

## Data Availability

The data that supports the findings of this study are presented in the manuscript and the supplementary material of this article.
