# Supplementary material for: TLR4 phosphorylation at tyrosine 672 activates the ERK/c‐FOS signaling module for LPS‐induced cytokine responses in macrophages
Source: Eur J Immunol. 2023 May 1;53(7):2250056. doi: 10.1002/eji.202250056 (PMC10947571; doi:10.1002/eji.202250056)
Supplement: Supplementary file 1 — Supporting information [file EJI-53-0-s001.pdf]

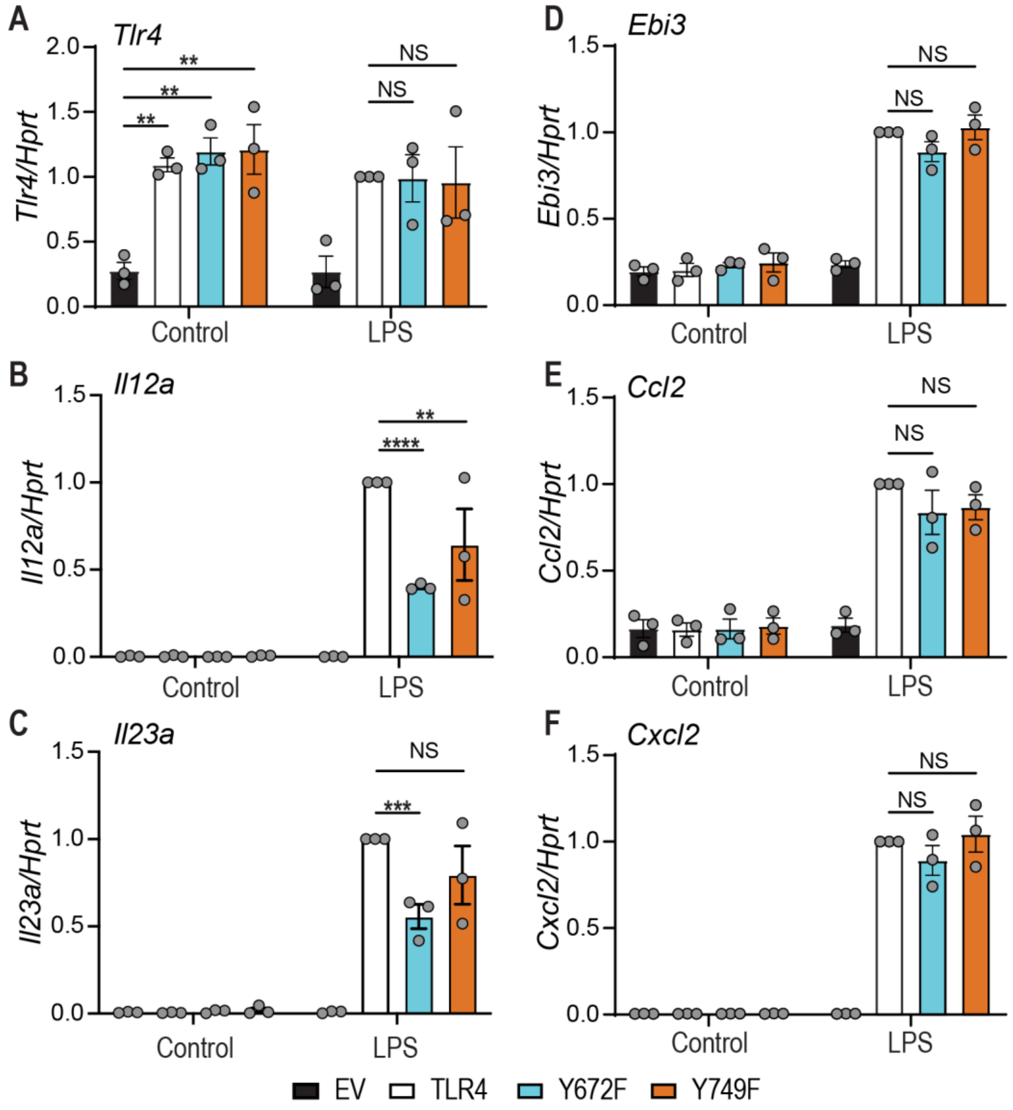

Supplementary Figure 1. Inflammatory gene expression in TLR4 Y672F and Y749F expressing BMM

A-F. *Tlr4*$^{-/-}$ BMM were reconstituted with wild type TLR4, TLR4 mutants or empty vector (EV). Cells were stimulated with LPS (1 ng/ml) for 4 h before total RNA was collected and assessed by RT-qPCR for mRNA levels of *Tlr4* (**A**), *Il12a* (**B**), *Il23a* (**C**), *Ebi3* (**D**), *Ccl2* (**E**) *and Cxcl2* (**F**). Data (mean+SEM, n=3) are combined from three independent experiments and are normalised to the wild type TLR4 + LPS sample. Statistical analyses were performed using a repeated measures two-way ANOVA, followed by Bonferroni's multiple comparison test (NS non-significant, ** $p<0.01$, *** $p<0.001$, **** $p<0.0001$).

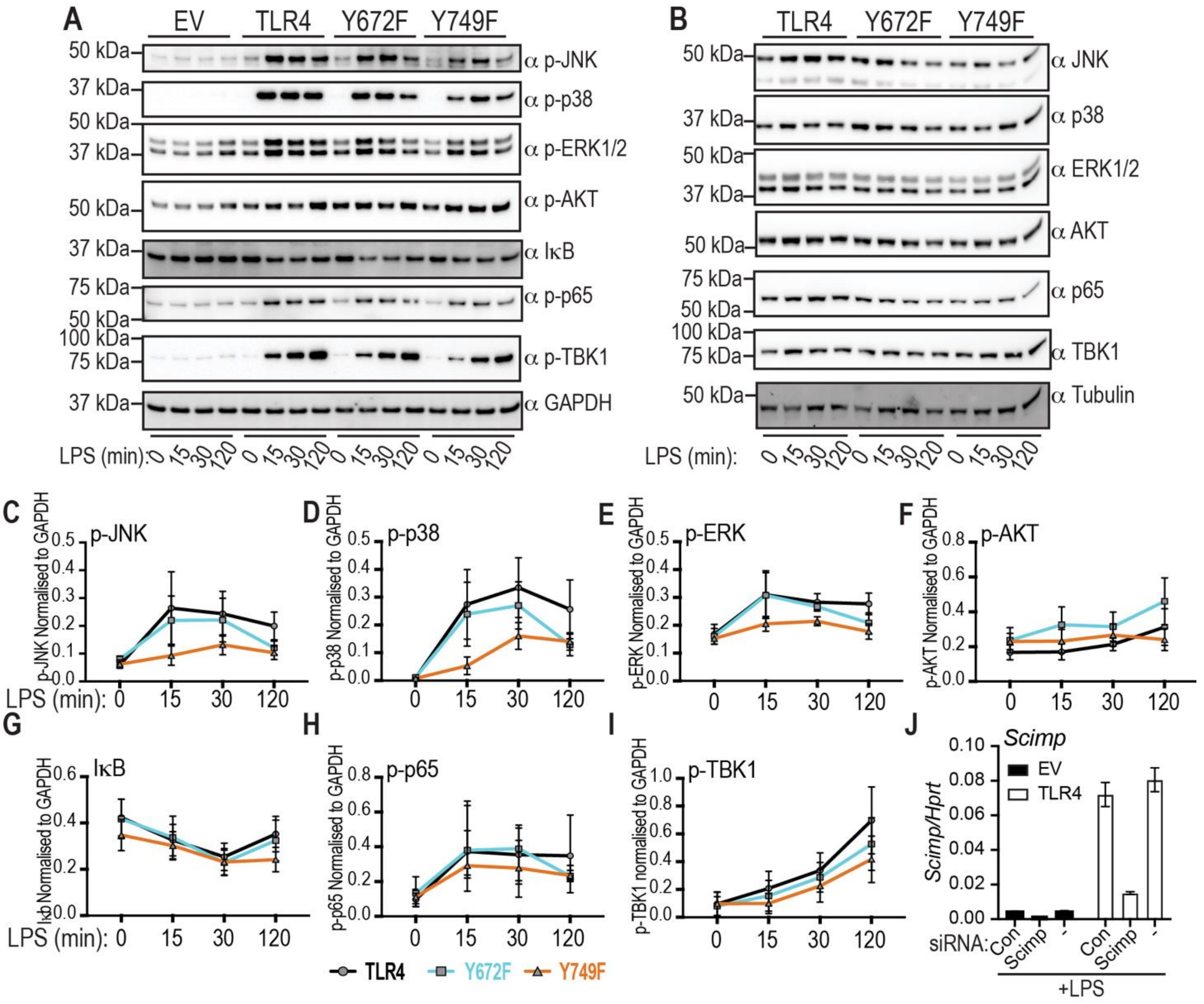

Supplementary Figure 2. LPS signaling responses in TLR4 Y672F- and TLR4 Y749-expressing BMM.

A-J. *Tlr4*$^{-/-}$ BMM were reconstituted with wild type TLR4, TLR4 mutants or empty vector (EV). **A-I.** Cells were then treated with LPS (1 ng/mL) for the indicated time points. Whole cell lysates were collected and assessed by western blot for levels of phosphorylated or total proteins, as indicated. **C-I.** Western blots were quantified for protein expression (relative to GAPDH expression levels). Data (mean + SEM, n=3-4) are combined from three to four independent experiments. **J.** Cells were electroporated with *Scimp* siRNA, a control siRNA or no siRNA, after which cells were treated with LPS (10 ng/mL) for 4 h before total RNA was collected and assessed by RT-qPCR for mRNA levels of *Scimp*. Data (mean + SD) are from one experiment.

Supplementary Table 1. Cloning primers

**Primer**	**Sequence (5'-3')**
Tlr4 Fwd	AAAATGCCAGGATGATGCCTCC
Tlr4 Rev	TCAGGTCCAAGTTGCCGTTTCT
Tlr4 NO STOP Rev	GGTCCAAGTTGC CGTTTCT
Tlr4 Y672F Fwd	GGAGAAAGCATCTTTGATGCATTT
Tlr4 Y672F Rev	ATTCTGACTCGAGAAGATCACA
Tlr4 Y749F Fwd	GTATCTTTGAATTTGAGATTGCTCA
Tlr4 Y749 Rev	TGAGCAATCTCAAATTCAAAGATAC
hTLR4 Y674A Fwd	GTATGGTAGAGGTGAAAACATCGCCGATGCCTTTGTTATCTAAAG
hTLR4 Y674A Rev	CTTGAGTAGATAACAAAGGCATCGGCGATGTTTCACCTCTACCATAC
hTLR4 Y751A Fwd	CGCTGGTGTATCTTTGAAGCCGAGATTGCTCAGACCTGG
hTLR4 Y751A Rev	CCAGGTCTGAGCAATCTCGGCTTCAAAGATACACCAGCG
hTLR4 Y674F Fwd	TGAAAACATCTTCGATGCCTTTGTTATC
hTLR4 Y674F Rev	CCTCTACCATACTTTATGC
hTLR4 Y751F Fwd	TATCTTTGAATTTGAGATTGCTCAGACCTGG
hTLR4 Y751F Rev	CACCAGCGGCTCTGGATG

Supplementary Table 2. Constructs

**Gene**	**Backbone**
Tlr4	pEF6
Tlr4 V5	pEF6
Tlr4 Y672F	pEF6
Tlr4 Y672F V5	pEF6
Tlr4 Y749F	pEF6
Tlr4 Y749F V5	pEF6
Tlr4	pMIGRMCS_GFP
Tlr4 V5	pMIGRMCS_GFP
Tlr4 Y672F	pMIGRMCS_GFP
Tlr4 Y672F V5	pMIGRMCS_GFP
Tlr4 Y749F	pMIGRMCS_GFP

Tlr4 Y749F V5	pMIGRMCS_GFP
hTLR4-mEGFP	pcDNA3
hTLR4 Y674A-*mEGFP*	pcDNA3
hTLR4 Y674F-*mEGFP*	pcDNA3
hTLR4 Y751A -*mEGFP*	pcDNA3
hTLR4 Y751F-*mEGFP*	pcDNA3

Supplementary Table 3. Antibodies

Antibody	Application	Dilution	Manufacturer/Catalogue
Mouse anti-V5 tag	WB	1:2500 (0.4 μg/mL)	Bio-Rad, California, USA Cat: MCA1360
Rabbit anti-GAPDH	WB	1:1000 (1 μg/mL)	R&D Systems, Minneapolis, USA Cat: RDS2275PC100
Rabbit anti-p-Jnk (Thr183/185)	WB	1:1000 (293 ng/mL)	Cell Signaling Technology, Boston, USA Cat: 4668
Rabbit anti-p-ERK1/2 (Thr 202/204)	WB	1:1000 (101 ng/mL)	Cell Signaling Technology, Boston, USA Cat: 9101S
Rabbit anti-p-p38 (MAPK) (Thr180/182)	WB	1:1000 (46 ng/mL)	Cell Signaling Technology, Boston USA Cat: 9211
Rabbit anti-p-p65 (NFκB) (Ser 536)	WB	1:1000 (57 ng/mL)	Cell Signaling Technology, Boston, USA Cat: 3033
Rabbit anti-IκB	WB	1:1000 (325 ng/mL)	Cell Signaling Technology, Boston, USA Cat: 4812
Rabbit anti-p-AKT (Ser 473)	WB	1:1000 (10 ng/mL)	Cell Signaling Technology, Boston, USA Cat: 9271
Rabbit anti-p-TBK1 (Ser 172)	WB	1:1000 (141 ng/mL)	Cell Signaling Technology, Boston, USA Cat: 5483
Rabbit anti-p-cFOS (Ser 32)	WB	1:1000 (57 ng/mL)	Cell Signaling Technology, Boston, USA

			Cat: 5348
Rabbit anti-JNK	WB	1:1000<br>(1 μg/mL)	Cell Signaling Technology, Boston, USA<br>Cat: 9258
Rabbit anti-p38	WB	1:1000<br>(23 ng/mL)	Cell Signaling Technology, Boston, USA<br>Cat: 9212
Rabbit anti-ERK1/2	WB	1:1000<br>(19 ng/mL)	Cell Signaling Technology, Boston, USA<br>Cat: 9102
Rabbit anti-AKT	WB	1:1000<br>(31 ng/mL)	Cell Signaling Technology, Boston, USA<br>Cat: 9272
Rabbit anti-p65	WB	1:1000<br>(208 ng/mL)	Cell Signaling Technology, Boston, USA<br>Cat: 8242
Rabbit anti-TBK1	WB	1:1000<br>(174 ng/mL)	Cell Signaling Technology, Boston, USA<br>Cat: 3013
Mouse anti-phosphotyrosine (4G10)	WB	1:1000	Merck, Darmstadt, Germany<br>Cat: 05-1050
Goat HRP-linked anti-rabbit IgG	WB	1:2500<br>(24 ng/mL)	Cell Signaling Technology, Boston, USA<br>Cat: 7074
Goat HRP-linked anti-mouse IgG	WB	1:2500<br>(73.6 ng/mL)	Cell Signaling Technology, Boston, USA<br>Cat: 7076
Human- Rhodamine Anti-GAPDH	WB	1:10000	Bio-Rad, California, USA<br>Cat: 12004167
Rat anti- mouse IL-6<br>(Capture)	ELISA	1:500<br>(1 μg/mL)	BD Biosciences, California, USA<br>Cat: 554400
Rat biotinylated anti- mouse IL-6<br>(Detection)	ELISA	1:500<br>(1 μg/mL)	BD Biosciences, California, USA<br>Cat: 554402
Rat anti- mouse IL-12p40<br>(Capture)	ELISA	1:250<br>(4 μg/mL)	BD Biosciences, California, USA<br>Cat: 551219

Rat biotinylated anti- mouse IL-12p40 (Detection)	ELISA	1:500<br>(2 μg/mL)	BD Biosciences, California, USA<br>Cat: 554476
Rat anti- mouse TNF<br>(Capture)	ELISA	1:250	BD Biosciences, California, USA<br>Cat: 558534
Rat biotinylated anti- mouse TNF<br>(Detection)	ELISA	1:250	BD Biosciences, California, USA<br>Cat: 558534
Rat APC-conjugated anti-mouse TLR4 (SA 15-21)	Flow	2.5 μg/mL	Biolegend<br>Cat: 145406

Supplementary Table 4. RT-qPCR primers

**Gene**	**Forward Primer (5'-3')**	**Reverse Primer (5'-3')**	**Amplicon size (bp)**
Il6	CTGCAAGAGACTTCCATCCAGTT	GAAGTAGGGAAGGCCGTGG	70
Il12b	GGAAGCACGGCAGCAGAATA	AACTTGAGGGAGAAGTAGGAATGG	180
Tnf	CATCTTCTCAAAATTCGAGTGACAA	TGGGAGTAGACAAGGTACAACCC	175
Ifnb1	CCACAGCCCTCTCCATCAAC	TGAAGTCCGCCCTGTAGGTG	123
Hprt	GCAGTACAGCCCCAAAATGG	AACAAAGTCTGGCCTGTATCCAA	85
Tlr4	TGGCTAGGACTCTGATCATGGC	TGAAGAAGGAATGTCATCAGGG	143
Ccl2	GCTTCTTTGGGACACCTGCTG	CCCACTCACCTGCTGCTACTCA	99
Ebi3	GACGTGCACCTGTTCTCCAC	GATGATTCGCTCAGCCACAA	105
Cxcl2	GAACAAAGGCAAGGCTAACTGAC	CATCAGGTACGATCCAGGCTTC	120
Il12a	GGTGAAGACGGCCAGAGAAA	TCTTCAATGTGCTGGTTTGGTC	98
Il23a	CCAGCGGGACATATGAATCTACT	ACAACCATCTTCACACTGGATACG	87
